# Influence of Different Hot Air Drying Temperatures on Drying Kinetics, Shrinkage, and Colour of Persimmon Slices

**DOI:** 10.3390/foods9010101

**Published:** 2020-01-18

**Authors:** Wijitha Senadeera, Giuseppina Adiletta, Begüm Önal, Marisa Di Matteo, Paola Russo

**Affiliations:** 1 School of Mechanical and Electrical Engineering, Faculty of Health, Engineering and Sciences, Springfield Campus, University of Southern Queensland, 37 Sinnathamby Boulevard, Springfield Central, QLD 4300, Australia; 2 Department of Industrial Engineering, University of Salerno, Via Giovanni Paolo II132, 84084 Fisciano, SA, Italy; bonal@unisa.it (B.Ö.); mdimatteo@unisa.it (M.D.M.); 3 Department Chemical Engineering Materials Environment, Sapienza University of Rome, Via Eudossiana, 18, 00184 Rome, Italy; Paola.Russo@uniroma1.it

**Keywords:** persimmon, “Rojo Brillante”, hot air drying, shrinkage, empirical mathematical model, colour

## Abstract

Drying characteristics of persimmon, cv. “Rojo Brillante”, slabs were experimentally determined in a hot air convective drier at drying temperatures of 45, 50, 55, 60, and 65 °C at a fixed air velocity of 2.3 m/s. It was observed that the drying temperature affected the drying time, shrinkage, and colour. Four empirical mathematical models namely, Enderson and Pabis, Page, Logarithmic, and Two term, were evaluated in order to deeply understand the drying process (moisture ratio). The Page model described the best representation of the experimental drying data at all investigated temperatures (45, 50, 55, 60, 65 °C). According to the evaluation of the shrinkage models, the Quadratic model provided the best representation of the volumetric shrinkage of persimmons as a function of moisture content. Overall, higher drying temperature (65 °C) improved the colour retention of dried persimmon slabs.

## 1. Introduction

The persimmon (*Diospyros kaki*) belongs to the family Ebenaceae and it is commonly cultivated in warm regions of the world including China, Korea, Japan, Brazil, Spain, Turkey, Italy, and Israel [1,2]. “Rojo Brillante” is one of the main cultivars produced in the Mediterranean area, including Italy. Among the other and known persimmon cultivars, the “Rojo Brillante” is the most popular cultivar due to its high productivity and commercial quality [3,4]. This persimmon cultivar is seedless and ripens faster than the other cultivars. In addition, persimmon fruits contain large amounts of bioactive compounds, such as ascorbic acid, carotenoids, and condensed tannins with strong antioxidant activity, which also offer health promoting effects (i.e., anticarcinogenic, anti-inflammatory, cardioprotective, and anti-hypercholesterolemic) [1,5,6,7]. Furthermore, persimmon fruits have a high content of sugars, as glucose and fructose, and moisture. For this reason, after the harvesting process, the fruits decay rapidly and they become very sensitive to microbial spoilage during storage.

These undesirable adverse changes may result in low quality and short shelf life. Due to these reasons, feasible processing and preservation technologies have been proposed to extend the shelf life of fresh fruits, reduce economic and environmental losses, valorise the traditional products, and increase their commercial value [3,6,8,9].

The persimmons are consumed in different forms, for example, fresh, frozen, canned, as well as their dehydrated form, and they can be stored for up to 6 month in a controlled or modified atmosphere. Dried persimmons have become an interesting product for consumers and global markets because they can be a valuable ingredient in different kinds of preparations including breakfast cereals, muesli, and snacks [1,3,10].

Drying is widely applied to fresh products as a preservation technique. The dehydration process prolongs the fruits and vegetables’ stability by reducing the water content and microbial growth and minimizing physicochemical changes. In addition, this process provides a better preservation of high-value compounds of foodstuffs; prolongs shelf life; reduces packaging, storage, and transportation costs due to the decreased food product weight and volume; and allows for the possibility of persimmon consumption during all seasons [1,6,9,11]. Whole persimmons have been used traditionally for the dehydration process to obtain a product with good sensory properties, however, drying of whole persimmon fruits is difficult. Using smaller persimmon fruits could be an alternative application to reduce drying time [3,12]. Fruits and vegetables are usually dehydrated in sun light, a solar dryer, or in artificial dryers [13]. Another food drying technique is conventional hot air drying, which is well-known as a cheap method, offers hygiene, uniformity, simplicity, ease of handling, affordability, and it provides better dried food materials [6,9,14,15,16].

Mathematical modelling in fruit drying is crucial to estimate optimal drying process conditions for prolonging the shelf life of food materials. Mathematical models of the drying process are applied for designing and improving industrial drying systems to obtain high quality dried products [1,17].

Shrinkage of fruits and vegetables is a widely known physical phenomenon during the drying process and it affects the overall quality of dried foodstuffs. This negative phenomenon leads to volume reduction, changes in shape and porosity, hardness increase, and surface cracking. It may also modify the microstructure and change the heat and mass transfer and rehydration capabilities of dried fruits. From this viewpoint, the shrinkage phenomenon has to be avoided since this undesirable physical change may cause a negative impression on consumers [9,17,18].

The main objective of this research was to investigate the effects of air drying conditions (temperature and time) on the drying behaviour and colour of persimmon, cv. “Rojo Brillante”, slabs. Therefore, we decided to use a wide range of drying temperatures (from 45 to 65 °C) to deeply evaluate the drying process variables and achieve high quality dried persimmon snacks. Furthermore, to describe the observed changes in water content during the drying tests, the moisture ratios were fitted using empirical models found in the literature. The drying models used in this research can be very important tools to estimate the persimmon slabs’ behaviour under different drying conditions and to optimize the drying process.

A good knowledge of the shrinkage phenomenon and the impact of process parameters on the mechanism of shrinkage are necessary to predict the shrinkage behaviour of fruits and vegetables. According to our knowledge, no scientific studies have been published related to the effect of the drying process on shrinkage of persimmon fruits nor the evaluation of shrinkage, including the mathematical models. To take into account the shrinkage effect on the quality of persimmon slabs during the drying process, volume changes were also measured and some empirical models of shrinkage were tested to describe the shrinkage behaviour during drying.

We believe that this research will contribute to the literature by providing a better understanding of the shrinkage behaviour of persimmon slices, how to control and optimise the drying process conditions, and how to obtain nutritious dried persimmon slabs for the dried fruit market.

## 2. Materials and Methods

### 2.1. Raw Materials

**Figure 1:**
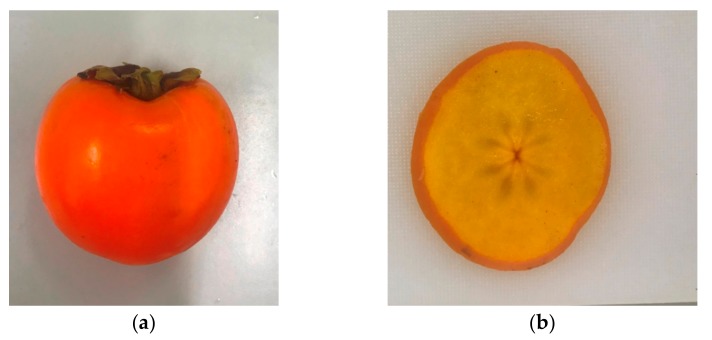
(**a**) Whole persimmon fruit, cv. “Rojo Brillante”; (**b**) Internal view of the persimmon fruit, cv. “Rojo Brillante”.

Persimmon fruits, cv. “Rojo Brillante”, were harvested from ten trees at the ripening stage at a commercial orchard located in Francolise (Caserta-Italy). Fresh whole fruits were washed, peeled, and sliced (Figure 1). Slices of a cylindrical shape (diameter of 30 mm and thickness of 6 mm) were prepared from the internal part of the fruits, without seeds, using a suitable steel mould. Before the experiments, all slabs were collected from different peeled persimmons. Sample randomization was performed to avoid undesirable differences in the structure of persimmons that could negatively affect the analysis.

### 2.2. Drying Experiments

Drying experiments of persimmon slabs were conducted in a convective dryer (FCV/E6L3, Zanussi, Pordenone, Italy) operating at a constant temperature. The dryer is comprised of a stainless-steel chamber (86 cm × 86 cm × 76 cm) equipped with an electric heater to heat the air and a centrifugal fan to supply the air flow and re-circulate the air.

The persimmon slabs were put on a plastic grid of mesh of size 0.01 m × 0.01 m in the dryer, and dried at 45, 50, 55, 60, and 65 °C at a centrifugal fixed air velocity of 2.3 m/s until the mass was constant (about 0.04 kg water/kg db). For drying kinetics, at suitable time intervals, some slices were taken out of the oven to calculate weight loss.

Weight loss was measured by means of an external digital electronic balance (mod. E42, Gibertini, Milano, Italy). The procedure was repeated until the mass of the sample no longer changed. For each temperature, drying tests were repeated in three sets independently. Each set had three replicates, and the averages with standard deviations are shown.

The results were reported as moisture ratio (*M_t_*/*M*_0_) vs. time (min), where *M_t_* was the moisture content (kg water/kg db) at a certain drying time and *M*_0_ (kg water/kg db) was its initial value [9].

### 2.3. Modelling of Drying Kinetics

Simplified drying models have been applied for describing the drying kinetics of several food products. Four empirical mathematical models widely used for fruits were here utilized (Table 1) to find the most appropriate model to describe the drying behaviour.

The empirical constants for the drying models were obtained from normalized experimental drying data (moisture ratio *M_t_*/*M*_0_ vs. time) at each investigated temperature. Nonlinear least square regression analysis was applied for the determination of the selected models’ parameters with the Levenberge–Marquardt procedure. For each model, the goodness of fit was assessed based upon the values of the following statistical parameters: the coefficient of determination (*R*^2^), the root mean square error (RMSE), and the reduced χ-square (χ^2^) [1,8,20,22].

These parameters were calculated as follows:

(1) $$\mathrm{RMSE}={\left[\frac{1}{N}\sum_{i=1}^{N}{\left(M_{R,pre,i}-M_{R,exp,i}\right)}^{2}\right]}^{1/2}\mathrm{and}$$

(2) $$\chi^{2}=\frac{\sum_{i=1}^{N}{\left(M_{R,pre,i}-M_{R,exp,i}\right)}^{2}}{N-z},$$

where *M_R,exp,i_* and *M_R,pre,i_* are experimental and predicted dimensionless moisture ratios, respectively, *N* is the number of observations, and *z* is the number of constants. The χ^2^ is the mean square of the deviations between the experimental and calculated values for the models. The lower the value of χ^2^, the better the goodness of the fit. The RMSE explains the deviation between the predicted and experimental values and it is necessary to reach zero.

The *R*^2^ was used as the primary comparison criteria for choosing the best model to consider the variation in the drying curves of dried fruits [1]. Its value should be higher and close to one. In addition to *R*^2^, χ^2^ and RMSE parameters were used to determine the quality of the fit [1,8,22]. The higher the value of *R*^2^, the lower the values of χ^2^ and RMSE, which were chosen as the criteria for goodness of fit [1,8,19].

### 2.4. Colour Evaluation

Surface colour was determined by two readings on the two different symmetrical faces of the fresh and dried persimmon slices using a Minolta Chroma Meter II Reflectance CR-300 colorimeter (Minolta, Osaka, Japan). The instrument was calibrated with an international standard white calibration plate CR-A43.

The repetitions of colour measurements were carried out in three sets independently for each temperature. Each set had six slices, and the averages of results were expressed with standard deviation.

CIE L*a*b* colour parameters (L*, a*, and b*) were measured for all samples and the average values were calculated. The lightness parameter (L*) represents the lightness/darkness of the persimmon samples, a* and b* parameters indicate the redness/greenness and yellowness/blueness of samples, respectively.

The Hue angle (H°) is how we perceive the colour of an object: green, orange, red, or blue; it was computed using the following equation [23]:

(3) $$H=tan^{-1}\frac{b^{*}}{a^{*}}$$

The total colour difference (ΔE) was calculated according to Equation (4) [9]:

(4) $$\Delta E\;=\;\sqrt[{2}]{\;{\left(\Delta L^{*}\right)}^{2}+\;{\left(\Delta a^{*}\right)}^{2}\;+\;{\left(\Delta b^{*}\right)}^{2}}$$

The fresh persimmon slabs were used as a reference material, and higher values of ΔE indicated more colour change from the reference persimmons.

### 2.5. Shrinkage Evaluation and Empirical Models

The initial volume of each persimmon (*V*_0_) was determined by using a digital Vernier caliper (0.01 mm accuracy), and it was calculated from diameter and thickness measurements for each slab (about 20 slices). The thickness and diameter dimensions were measured on the same slabs at specific times during drying tests, and the volume (*V_t_*) was calculated. Furthermore, the diameter and the thickness were measured at different sample positions to minimize the measurement error during drying, and their average values were estimated. The evaluation of shrinkage during drying was studied in terms of the mean volume shrinkage (*V_t_*/*V*_0_) reported as a function of the relative moisture ratio (*M_t_*/*M*_0_) [24].

An empirical correlation between shrinkage and moisture content can be used to model shrinkage during the drying process. Many empirical models are available in the literature and they are widely applied for vegetables and fruits [8,25,26]. The mathematical models taken for identifying the most suitable model to describe the shrinkage behaviour are reported in Table 2. Nonlinear least square regression analysis was used to evaluate the parameters of the selected model with the Levenberge–Marquardt procedure.

### 2.6. Statistical Analysis

The means of experimental results and their standard deviations were calculated from three replicates. One-way analysis of variance (ANOVA) using Tukey’s test (*p* < 0.05) was conducted to compare the means in the case of colour.

## 3. Results and Discussion

### 3.1. Drying Kinetics: Experiments and Empirical Models

**Figure 2:**
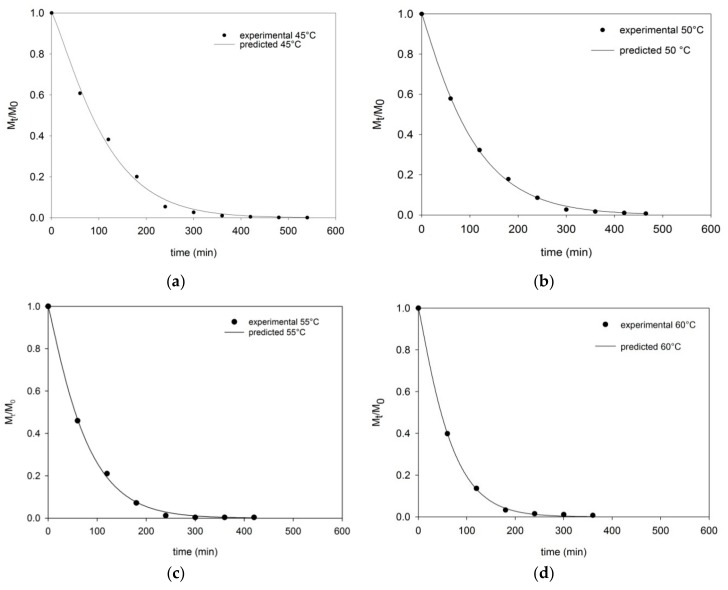

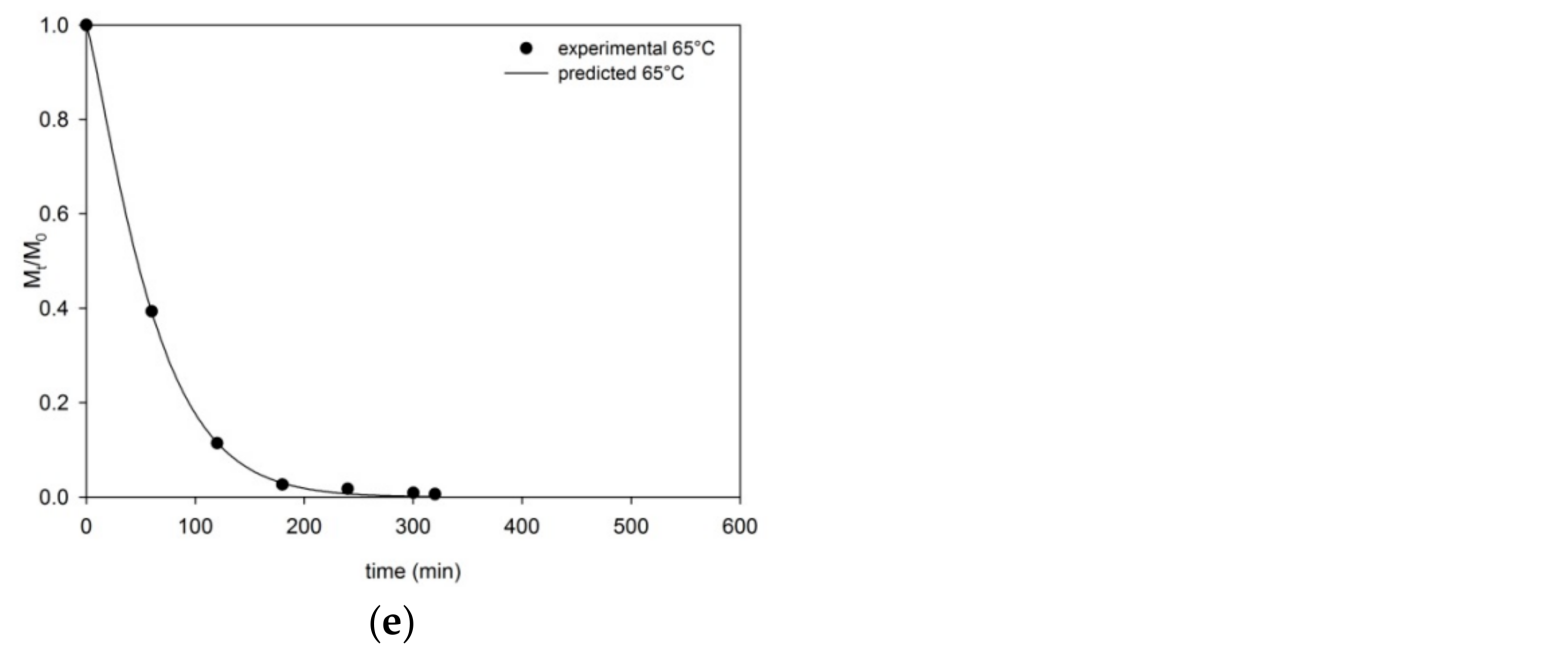
Experimental (symbols) and predicted (lines) drying curves in terms of moisture ratio *(M_t_*/*M*_0_) of persimmon samples at (**a**) 45 °C, (**b**) 50 °C, (**c**) 55 °C, (**d**) 60 °C, and (**e**) 65 °C

The average moisture content of fresh persimmon fruits was 5.23 ± 0.19 g water/g db (83.94% wb). To evaluate the impact of different drying temperatures (45–65 °C) on persimmon drying kinetics, the curves of moisture ratio *M_t_*/*M*_0_ vs. drying time (min) are presented in Figure 2a–e. It was clear that the moisture content decreased with increased drying time. As shown in Figure 2, the changes in moisture content at all investigated temperatures were more evident in the first drying stage; while at the final stage these changes became very small.

The drying times of all samples needed to achieve an equilibrium moisture content (<0.05 kg water/kg db) were 540, 465, 420, 360, and 320 min at 45, 50, 55, 60, and 65 °C, respectively. From the investigated temperature range, as expected, these results showed that the drying time is the longest at 45 °C and shortest at 65 °C. In order to predict the water content as a function of drying time, the empirical equations, presented in Table 1, were fitted and statistical parameters and estimated model parameters are shown in Table 3 and Table 4, respectively.

The coefficient of determination (*R*^2^), the reduced χ-square (χ^2^), and the root mean square error (RMSE) were used to describe the quality of the fit (Table 3). A good fitting among the experimental and theoretical data was connected to the highest *R*^2^ value and the lowest χ^2^ and RMSE values.

All *R*^2^ values of Enderson and Pabis, Page, Logarithmic, and Two term models were higher than 0.99, while χ^2^ and RMSE ranged from 0.0001 to 0.0015 and 0.0069 to 0.0382, respectively.

The models’ parameters are presented in Table 4. For Enderson and Pabis, Page, and Logarithmic models, the drying constant k had a value of 0.003 to 0.016 and it increased with an increase in drying air temperature. Furthermore, for the Two term model, the drying constants (k_1_ and k_2_) had a value of 0.009 to 0.017 and these values increased with an increase in drying temperature.

From the models’ results, the Page model was found to be the most appropriate model to describe the persimmon drying curves at all investigated temperatures, with the highest *R*^2^ values and the lowest χ^2^ and RMSE values. On the other hand, the Two term model had the worst fitting for persimmon slabs dried in the range 45–65 °C.

The experimental data and the results of the best-fitting model (Page model) are shown in Figure 2a–e. The Page model was able to predict with sufficient accuracy the evolution of moisture content for persimmon slabs at each drying temperature.

Doymaz [1] stated the suitability of the Page model to fit the experimental drying data of persimmon slices in comparison with other empirical models at 50, 60, and 70 °C.

### 3.2. Colour Evaluation

Colour is well known as one of the most important quality parameters of fresh and dried fruits. It is widely utilized as a tool for foodstuff standardization, indicator of biological and/or physicochemical traits, quality properties, as well as consumer satisfaction. Unsuitable changes in colour of fruits and vegetables affect their quality and marketing value [27]. Colour is also a fundamental quality parameter in food choice and it influences the perception of the other sensorial attributes by consumers [3]. The effect of air drying temperatures on colour characteristics of fresh and dried persimmon samples are presented in Table 5 in which L*, a*, and b* value, Hue angle (H°), and total colour difference (ΔE) are presented. According to L* values, although the fresh persimmons had the highest L* values (71.70 ± 0.52), no significant differences (*p* > 0.05) were found between fresh and all dried persimmon slabs. This means that the drying process, particularly, air drying temperature, did not influence the lightness of the dried samples. There was not a remarkable reduction in brightness of persimmon fruits after the drying process.

As shown in Table 5, the a* values of samples were affected by the drying conditions (temperature and time). Concerning the dehydrated samples, a* values increased after the drying process. The highest a* values were found in persimmon slabs dried at 45 °C (5.93 ± 1.51) and 50 °C (5.61 ± 0.31). These colour changes of redness (a* value) may be associated to browning reactions due to long drying times at low temperatures. There were no statistical differences (*p* < 0.05) in b* values observed among all dried persimmon slices.

The derived indices from colour Hunter values (L*, a*, and b*), namely, Hue angle and total colour differences gave more information concerning the colour degradation of fresh and dried persimmons [28]. The best quality of dried persimmons may be correlated to low values of overall colour change (ΔE), which has an important role on the consumers’ acceptability.

Furthermore, H° values of dried samples were different from fresh persimmon fruit (*p* < 0.05); the persimmon samples dried at 65 °C showed higher Hue angle values in comparison with the other dried samples.

The drying process had a significant effect on the total colour differences of dried samples, and the lowest value of ΔE was found in the samples dried at 65 °C, indicating that the highest temperature (65 °C) could preserve the typical colour of the fresh persimmon fruits and contributed to reduced browning reactions during the drying process.

These results may be explained by the long exposure time to the drying process at low temperature (45 °C) and the enzymatic browning reaction that occurred during the persimmon drying process, since the temperature and the time of the drying are crucial factors leading to colour deterioration.

### 3.3. Shrinkage and Empirical Models

**Figure 3:**
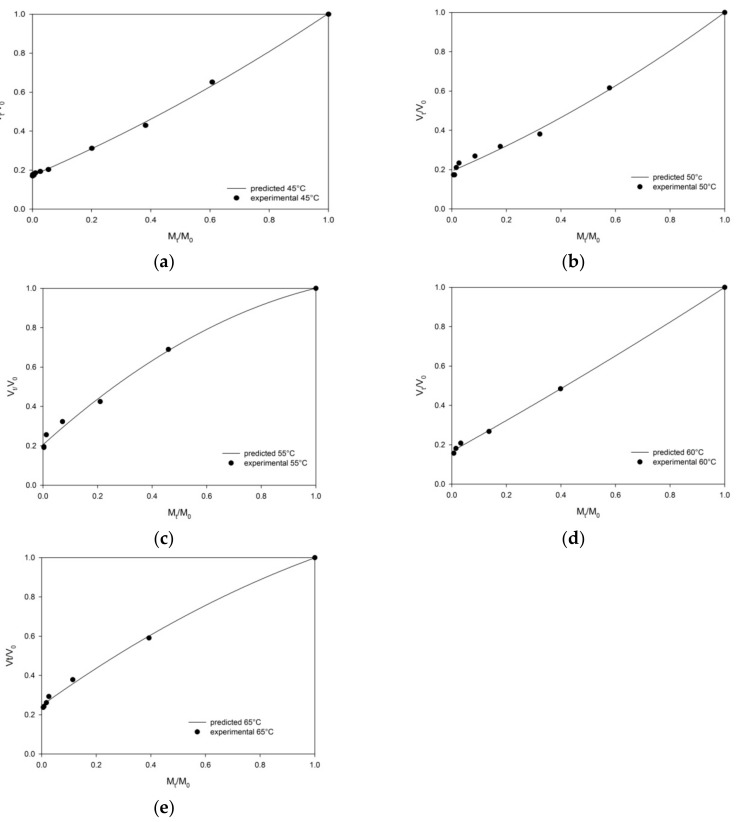
Experimental data (symbols) and prediction (curves) of volume shrinkage in terms of volume ratio (*V_t_*/*V*_0_) of persimmon samples during drying at (**a**) 45 °C, (**b**) 50 °C, (**c**) 55 °C, (**d**) 60 °C, and (**e**) 65 °C.

The shrinkage phenomenon is known as one of the most important physical changes that negatively impacts the quality of the dried foodstuffs [9,25]. The effects of different air drying temperatures on the shrinkage of persimmon slabs’ were evaluated and the changes in volume ratio (*V_t_*/*V*_0_) as a function of the moisture ratio (*M_t_*/*M*_0_) are presented in Figure 3a–e.

A reduction of persimmon volume proportional to the moisture content decrease during the drying process at all evaluated temperatures was found. The lower the shrinkage, the higher the drying temperatures (60–65 °C).

For the Linear, Quadratic, and Exponential models, which correlated shrinkage and moisture content, the statistical coefficients (*R*^2^ and RMSE) and the estimated model parameters are reported in Table 6 and Table 7, respectively.

The *R*^2^ values of the Linear, Quadratic, and Exponential models were all above 0.90. The estimations of statistical parameters demonstrated that *R*^2^ and RMSE values ranged from 0.8992 to 0.99898, and 0.0103 to 0.1002, respectively (Table 6). The nonlinear model (Quadratic model) predicted the changes in the shrinkage of the persimmon slices significantly better than did the Linear and Exponential models for all drying conditions. Under the most ideal condition, the shrinkage is expressed as a linear function of the moisture ratio where a_1_ and a_2_ are coefficient and constant, respectively, of the model. On the contrary, in this study, the Linear model was found to be an inappropriate model for describing the persimmon shrinkage vs. moisture ratio at all investigated temperatures. Also, the volume ratio *V_t_*/*V*_0_ and moisture ratio *M_t_*/*M*_0_ had a poor exponential relationship for all hot air dried persimmon slabs, with the lowest value of *R*^2^ (0.899) at 55 °C and the lowest value of the slope of this model, k, at 65 °C (Table 7).

The Quadratic model was the best model to describe the volumetric shrinkage of all the persimmon samples as a function of moisture content at all the drying temperatures investigated, with the highest *R*^2^ values and the lowest RMSE values.

These finding, in other words, the Quadratic model results for volumetric shrinkage with moisture ratio, were similar to those reported by Adiletta et al. [8] and Seerangurayar et al. [18] for convective air dried grapes and solar dried dates, respectively. Furthermore, Mayor and Sereno [25] found that the Quadratic model had a good fit to experimental data of volumetric shrinkage vs. moisture ratio for apples, carrots, and potato slabs during convective drying.

In order to verify the proposed model, experimental and predicted shrinkage data are plotted in Figure 3 as a function of moisture ratio. It is clear that in the Quadratic model the predicted data had a good agreement (*R*^2^ > 0.994) with the experimental data.

## 4. Conclusions

The effect of the drying process conditions on drying characteristics and on the colour of persimmon, cv. “Rojo Brillante”, slabs was investigated in this study. As expected, the drying process was shorter at higher drying temperatures. The experimental drying data were fitted to four empirical mathematical models, and the Page model described the best representation of the experimental drying values at all investigated temperatures (45, 50, 55, 60, and 65 °C). Furthermore, concerning the shrinkage phenomenon, the Quadratic model demonstrated an acceptable fit to the experimental data for all dried persimmon samples. Persimmon samples dried at 65 °C showed better colour preservation in terms of the higher Hue angle (H°) value and less total colour change (ΔE). The findings of this research may be important by providing information for understanding the drying behaviour and the drying process conditions of persimmon slabs from an industrial perspective.

## Figures and Tables

**Table 1:** Mathematical models applied to drying curves.

Model Name	Equation	Reference
Henderson and Pabis	$\frac{M_{t}}{M_{0}}=a\;exp\left(-kt\right)$	Henderson and Pabis [19] (1961); Adiletta et al. [8] 2016
Page	$\frac{M_{t}}{M_{0}}=exp\left(-kt^{n}\right)$	Doymaz [1] (2012); Adiletta et al. [8] 2016
Logarithmic	$\frac{M_{t}}{M_{0}}=a\;exp\left(-kt\right)+c$	Yagcioglu et al. [20] (1999)
Two term	$\frac{M_{t}}{M_{0}}=a_{1}\;exp\left(-k_{1}t\right)+a_{2}\;exp\left(-k_{2}t\right)$	Henderson [21] (1974); Adiletta et al. [22] 2018

**Table 2:** Shrinkage models.

Model Name	Equation	References
Linear	$\frac{V_{t}}{V_{0}}=a_{1}+a_{2}\left(\frac{M_{t}}{M_{0}}\right)\;$	Simal et al. [26]
Quadratic	$\;\frac{V_{t}}{V_{0}}=a_{1}+a_{2}\left(\frac{M_{t}}{M_{0}}\right)+a_{3}{\left(\frac{M_{t}}{M_{0}}\right)}^{2}\;$	Mayor and Sereno [25]
Exponential	$\;\frac{V_{t}}{V_{0}}=a_{1}exp\left(k\frac{M_{t}}{M_{0}}\right)\;$	Mayor and Sereno [25]

**Table 3:** Statistical parameters (coefficient of determination *R*^2^, the root mean square error RMSE, the reduced χ-square χ^2^) of the drying models.

Model Name	Parameters	Temperatures (°C)				
		45°	50°	55°	60°	65°
Henderson and Pabis	*R* ^2^	9.9 × 10^−1^	1.0 × 10^0^	1.0 × 10^0^	1.0 × 10^0^	1.0 × 10^0^
	RMSE	3.3 × 10^−2^	1.5 × 10^−2^	1.6 × 10^−2^	1.4 × 10^−2^	1.8 × 10^−2^
	χ^2^	1.1 × 10^−3^	3.0 × 10^−4^	3.0 × 10^−4^	2.0 × 10^−4^	3.0 × 10^−4^
Page	*R* ^2^	1.0 × 10^0^	1.0 × 10^0^	1.0 × 10^0^	1.0 × 10^0^	1.0 × 10^0^
	RMSE	2.3 × 10^−2^	9.3 × 10^−3^	1.2 × 10^−2^	6.9 × 10^−3^	7.1 × 10^−3^
	χ^2^	4.0 × 10^−4^	1.0 × 10^−4^	1.0 × 10^−4^	1.0 × 10^−4^	1.0 × 10^−4^
Logarithmic	*R* ^2^	9.9 × 10^−1^	1.0 × 10^0^	1.0 × 10^0^	1.0 × 10^0^	1.0 × 10^0^
	RMSE	2.8 × 10^−2^	1.1 × 10^−2^	1.4 × 10^−2^	1.5 × 10^−2^	1.9 × 10^−2^
	χ^2^	8.0 × 10^−4^	2.0 × 10^−4^	2.0 × 10^−4^	2.0 × 10^−4^	4.0 × 10^−4^
Two term	*R* ^2^	9.9 × 10^−1^	1.0 × 10^0^	1.0 × 10^0^	1.0 × 10^0^	1.0 × 10^0^
	RMSE	3.8 × 10^−2^	1.8 × 10^−2^	2.0 × 10^−2^	1.8 × 10^−2^	2.3 × 10^−2^
	χ^2^	1.5 × 10^−3^	4.0 × 10^−4^	4.0 × 10^−4^	3.0 × 10^−4^	5.0 × 10^−4^

**Table 4:** Model parameters (k, k_1_ and k_2_, the drying constants; a, a_1_, a_2_, c, n, the drying coefficients) of the drying models.

Model Name	Parameters	Temperatures (°C)				
		45°	50°	55°	60°	65°
Henderson and Pabis	a	1.0 × 10^0^	1.0 × 10^0^	1.0 × 10^0^	1.0 × 10^0^	1.0 × 10^0^
	k	9.2 × 10^−3^	9.7 × 10^−3^	1.4× 10^−2^	1.6 × 10^−2^	1.7 × 10^−2^
Page	k	3.2 × 10^−3^	6.0 × 10^−3^	8.0 × 10^−3^	8.8 × 10^−3^	6.7 × 10^−2^
	n	1.2 × 10^0^	1.1 × 10^0^	1.1 × 10^0^	1.1 × 10^0^	1.2 × 10^0^
Logarithmic	a	1.0 × 10^0^	1.0 × 10^0^	1.0 × 10^0^	1.0 × 10^0^	1.0 × 10^0^
	k	8.4 × 10^−3^	9.2 × 10^−3^	1.3 × 10^−2^	1.6 × 10^−2^	1.6 × 10^−2^
	c	−3.3 × 10^−2^	−2.0 × 10^−2^	−1.5 × 10^−2^	−6.1 × 10^−3^	−8.1 × 10^−3^
Two term	a_1_	5.2 × 10^−1^	5.1 × 10^−1^	5.1 × 10^−1^	5.1 × 10^−1^	5.1 × 10^−1^
	k_1_	9.2 × 10^−3^	9.7 × 10^−3^	1.4 × 10^−2^	1.6 × 10^−2^	1.7 × 10^−2^
	a_2_	5.0 × 10^−1^	5.0 × 10^−1^	5.0 × 10^−1^	4.9 × 10^−1^	4.9 × 10^−1^
	k_2_	9.2 × 10^−3^	9.7 × 10^−3^	1.4 × 10^−2^	1.6 × 10^−2^	1.7 × 10^−2^

**Table 5:** Colour parameters (lightness/darkness L*; redness/greenness a*; yellowness/blueness b*; Hue angle H°; total colour difference ΔE) for fresh and dried persimmon samples.

Sample	Drying Time (min)	L*	a*	b*	H°	ΔE
fresh persimmon		71.7 ± 0.5 ^a^	−1.6 ± 1.3 ^a^	46.4 ± 1.1 ^a^	92.0 ± 1.6 ^c^	-
persimmon dried at 45 °C	540	67.8 ± 1.8 ^a^	5.9 ± 1.5 ^d^	53.6 ± 2.0 ^b^	83.6 ± 0.0 ^a^	18.9 ± 1.1 ^c^
persimmon dried at 50 °C	465	68.7 ± 2.4 ^a^	5.6 ± 0.3 ^c,d^	53.3 ± 1.1 ^b^	84.0 ± 0.4 ^a^	13.7 ± 0.7 ^b^
persimmon dried at 55 °C	420	67.4 ± 2.5 ^a^	2.6 ± 1.1 ^b^	57.7 ± 2.4 ^b^	87.4 ± 1.1 ^a,b^	12.8 ± 1.0 ^b^
persimmon dried at 60 °C	360	70.5 ± 1.9 ^a^	3.0 ± 0.9 ^b,c^	56.2 ± 1.1 ^b^	87.0 ± 1.0 ^a,b^	12.1 ± 0.7 ^b^
persimmon dried at 65 °C	320	70.3 ± 0.8 ^a^	1.9 ± 0.8 ^b^	55.7 ± 4.0	88.0 ± 1.6 ^b^	9.2 ± 0.4 ^a^

Different superscript letters (a,b,c) in the same column show statistical differences between samples (*p* < 0.05).

**Table 6:** Statistical coefficients (*R*^2^, RMSE) of the shrinkage models.

Model Name	Parameters	Temperatures				
		45	50	55	60	65
Linear	*R* ^2^	1.0 × 10^0^	9.9 × 10^−1^	9.7 × 10^−1^	1.0 × 10^0^	9.9 × 10^−1^
	RMSE	2.0 × 10^−2^	3.0 × 10^−2^	5.2 × 10^−2^	1.3 × 10^−2^	2.9 × 10^−2^
Quadratic	*R* ^2^	1.0 × 10^0^	9.9 × 10^−1^	9.9 × 10^−1^	1.0 × 10^0^	1.0 × 10^0^
	RMSE	1.0 × 10^−2^	2.4 × 10^−2^	2.7 × 10^−2^	1.3 × 10^−2^	1.9 × 10^−2^
Exponential	*R* ^2^	9.7 × 10^−1^	9.8 × 10^−1^	9.0 × 10^−1^	9.8 × 10^−1^	9.5 × 10^−1^
	RMSE	4.9 × 10^−2^	4.4 × 10^−2^	1.0 × 10^−1^	5.7 × 10^−2^	6.7 × 10^−2^

**Table 7:** Model parameters (k, the shrinkage constant; a_1_, a_2_, a_3_, the shrinkage coefficients) of the shrinkage models.

Model Name	Parameters	Temperatures				
		45	50	55	60	65
Linear	a_1_	1.6 × 10^−1^	1.8 × 10^−1^	2.3 × 10^−1^	1.6 × 10^−1^	2.6 × 10^−1^
	a_2_	8.1 × 10^−1^	7.9 × 10^−1^	8.2 × 10^−1^	8.3 × 10^−1^	7.9 × 10^−1^
Quadratic	a_1_	1.6 × 10^−1^	1.9 × 10^−1^	2.1 × 10^−1^	1.7 × 10^−1^	2.5 × 10^−1^
	a_2_	6.5 × 10^−1^	6.0 × 10^−1^	1.2 × 10^0^	7.7 × 10^−1^	1.0 × 10^0^
	a_3_	1.8 × 10^−1^	2.1 × 10^−1^	−4.5 × 10^−1^	6.1 × 10^−2^	−2.4 × 10^−1^
Exponential	a_1_	2.1 × 10^−1^	2.1 × 10^−1^	2.7 × 10^−1^	2.1 × 10^−1^	2.9 × 10^−1^
	k	1.6 × 10^0^	1.6 × 10^0^	1.3 × 10^0^	1.6 × 10^0^	1.3 × 10^0^
